# NEIL3-mediated proteasomal degradation facilitates the repair of cisplatin-induced DNA damage in human cells

**DOI:** 10.1038/s41598-023-32186-3

**Published:** 2023-03-30

**Authors:** Umit Aliyaskarova, Yeldar Baiken, Flore Renaud, Sophie Couve, Alexei F. Kisselev, Murat Saparbaev, Regina Groisman

**Affiliations:** 1 Team «Mechanisms of DNA Repair and Carcinogenesis», CNRS UMR 9019, Université Paris-Saclay, Gustave Roussy Cancer Campus, 94805 Villejuif Cedex, France; 2 National Laboratory Astana, Nazarbayev University, Astana, Kazakhstan; 3 School of Sciences and Humanities, Nazarbayev University, Astana, Kazakhstan; 4 School of Engineering and Digital Sciences, Nazarbayev University, Astana, Kazakhstan; 5 EPHE, PSL University, Paris, France; 6 Department of Drug Discovery and Development, Harrison College of Pharmacy, Auburn University, PRB, 720 S. Donahue Dr., Auburn, AL 36849 USA

**Keywords:** Cancer therapeutic resistance, Chemotherapy, DNA damage and repair, Proteasome, Apoptosis

## Abstract

Anti-neoplastic effect of DNA cross-linking agents such as cisplatin, mitomycin C, and psoralen is attributed to their ability to induce DNA interstrand cross-links (ICLs), which block replication, transcription, and linear repair pathways by preventing DNA strand separation and trigger apoptosis. It is generally agreed that the Fanconi anemia (FA) pathway orchestrates the removal of ICLs by the combined actions of various DNA repair pathways. Recently, attention has been focused on the ability of the NEIL3-initiated base excision repair pathway to resolve psoralen- and abasic site-induced ICLs in an FA-independent manner. Intriguingly, overexpression of NEIL3 is associated with chemo-resistance and poor prognosis in many solid tumors. Here, using loss- and gain-of-function approaches, we demonstrate that NEIL3 confers resistance to cisplatin and participates in the removal of cisplatin–DNA adducts. Proteomic studies reveal that the NEIL3 protein interacts with the 26S proteasome in a cisplatin-dependent manner. NEIL3 mediates proteasomal degradation of WRNIP1, a protein involved in the early step of ICL repair. We propose that NEIL3 participates in the repair of ICL-stalled replication fork by recruitment of the proteasome to ensure a timely transition from lesion recognition to repair via the degradation of early-step vanguard proteins.

## Introduction

More than four decades after entering clinical practice, cisplatin remains one of the most widely used chemotherapeutic agents against a variety of solid tumors^1^. Nevertheless, the resistance of tumor cells to cisplatin reduces the clinical efficacy of this agent. Although it is known that the removal of cisplatin-induced DNA lesions is one of the main contributors to resistance^2^, the detailed mechanisms of repair are not fully understood^3,4^. The anti-tumor effect of cisplatin-based chemotherapy is attributed to its ability to induce DNA interstrand cross-links (ICLs), which can block both DNA replication and transcription, ultimately triggering apoptosis^5^. In mammals, the predominant and best-characterized mechanism of ICL repair is the Fanconi anemia (FA) pathway, which proceeds in a replication-coupled manner^6^. Moreover, alternative mechanisms of ICL repair that function independently or synergistically with the FA pathway have also been identified and characterized^7–9^.

NEIL3, together with NEIL1 and NEIL2, belongs to the family of Nei-like (NEIL) bifunctional DNA glycosylases. In the first step of the base excision repair (BER) pathway, they hydrolyze the glycosidic bond between the deoxyribose sugar and oxidized DNA base^10^. All three NEILs are bifunctional enzymes with overlapping DNA substrate specificity and an unusual preference for single-stranded DNA and other open structures that could be generated during DNA replication and transcription^11^. Unlike the other two members of the human NEIL DNA glycosylase family, NEIL3 has an extended C-terminal domain (CTD) that contains a RanBP2-like zinc finger (RanBP ZF) and two glycine-arginine-phenylalanine zinc fingers (GRF ZFs) motifs. These motifs mediate DNA–protein and protein–protein interactions^12–14^.

NEIL3-initiated BER is a major repair pathway and first-line defense against psoralen-induced ICLs^15^. Our laboratory was the first to demonstrate that, in addition to their classical oxidized DNA base excision activity, NEIL1 and NEIL3 resolve psoralen-induced ICLs in three- and four-stranded DNA structures^16–18^. Subsequently, Semlow and colleagues have shown that NEIL3 from *Xenopus laevis* can cleave the psoralen- and abasic site-induced ICLs in X-shaped dsDNA structures^12,19^. Loss of NEIL3 leads to decreased fork speed and increased DSB formation, whereas its overexpression in tumors is associated with genomic alterations and confers a poor prognosis^20,21^. Intriguingly, NEIL3 is overexpressed in highly proliferative tissues such as bone marrow and various tumors and is associated with metastasis in melanoma patients^22–24^. Although NEIL3 does not cleave the cisplatin-induced ICLs in Xenopus extracts, despite being recruited to this lesion^12,19^, NEIL3 knock-out cells are sensitive to cisplatin, suggesting a possible indirect role of NEIL3 in the repair of cisplatin-induced DNA adducts in mammalian cells^15,25^.

In mammalian cells, the degradation of the majority of proteins (at least 60%), including misfolded, damaged, no-longer-needed, and regulatory proteins, is mediated by the 26S proteasome. This multi-subunit proteolytic complex catalyzes the degradation of proteins modified by the covalent attachment of poly-ubiquitin chains. Each round of ubiquitin attachment occurs in 3 steps: first, the E1 enzyme activates the C-terminus of ubiquitin, a small 76 amino acids protein, in an ATP-dependent manner. Then, ubiquitin is transferred to the cysteine residue of an E2 enzyme. And finally, a specific E3 ubiquitin ligase recognizes a degradation signal in a substrate and mediates ubiquitin transfer from E2 to a lysine residue on the substrate and then to a ubiquitin molecule that is already attached to the substrate^26^. The 19S regulatory part of 26S proteasome recognizes and removes ubiquitin chains, unfolds, and translocates the substrate protein to the 20S core part, where it is degraded into small peptides^27,28^. The ubiquitin–proteasome pathway (UPP) regulates various cellular processes, including DNA replication and repair^27,29–31^. The proteasome is involved in homologous recombination, the Fanconi anemia pathway^32^, and nucleotide excision repair^33^.

In the present study, we demonstrate that the overexpression of exogenous NEIL3 protein dramatically improves the repair of cisplatin DNA lesions and protects cells from cisplatin-induced apoptosis. In contrast, the knockout of NEIL3 sensitizes cells to cisplatin and leads to the accumulation of cisplatin-induced DNA lesions. Proteomic analysis of the protein–protein interactions reveals that NEIL3 interacts with the 26S proteasome. Inhibition of the proteasome suppresses NEIL3-mediated cisplatin resistance, suggesting the role of protein degradation in DNA repair of cisplatin-induced DNA cross-links. We have identified WRNIP1, a member of the AAA ATPase family, as a possible protein target of NEIL3-dependent proteasomal degradation. Previously, it has been shown that WRNIP1 participates in the early step of ICL repair by recruiting FANCD2^34^. Here we propose a model in which WRNIP1 is rapidly recruited to the cisplatin-induced ICL-stalled replication fork in a complex with NEIL3 and then degraded in a timely manner at later stages of the repair.

## Results

### Gain- and loss-of-function approaches demonstrate the role of NEIL3 in cisplatin-induced apoptosis via DNA repair

**Figure 1:**
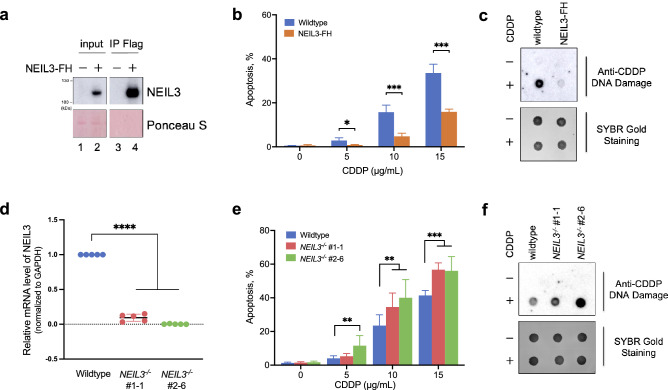
NEIL3 is involved in the repair of cisplatin-induced DNA lesions and protects human cells from cisplatin-induced apoptosis. (**a**) Western blot of Flag IP samples purified from HeLa S3 cells overexpressing NEIL3 protein (NEIL3-FH). Ponceau S staining was used as a loading control. Note that the membrane was cropped to remove irrelevant parts. (**b**) Measurement of apoptosis by flow cytometry of Annexin V and Propidium Iodide (PI) stained cells incubated overnight in the presence of different doses of cisplatin (CDDP). Data shown are the mean ± SD (n = 4). *p <0.05; **p <0.01; ***p <0.001. (**c**) Dot blot with an antibody recognizing cisplatin-modified DNA to measure the accumulation of cisplatin–DNA adducts after overnight exposure to cisplatin (15 µg/mL). SYBR Gold staining was used as a loading control. (**d**) Characterization of the HeLa S3 NEIL3-/- cell lines constructed using CRISPR-Cas9 genome editing technique. The disruption of the NEIL3 gene in clones #1–1 and #2–6 was confirmed by RT-qPCR. Data shown are the mean ± SD (n = 5). ****p <0.0001. (**e, f**) Same as in “b” and “c” panels except that HeLa S3 NEIL3^-/-^ cells were used. See Materials and Methods for additional details.

To study the emerging role of NEIL3 DNA glycosylase in cisplatin resistance, we first employed a gain-of-function approach. For this, we generated a HeLa S3 cell line with stable overexpression of NEIL3, tagged with Flag and HA epitopes at the C-terminus (NEIL3-FH) using retroviral transduction (Fig. 1a)^35^. Then HeLa S3 wildtype (WT) and NEIL3-FH cells were treated with different doses of cisplatin (0; 5; 10; and 15 µg/mL) overnight and stained with Annexin V and Propidium Iodide (PI) to evaluate apoptosis induction by flow cytometry. As a result, we observed significantly less apoptosis in NEIL3-FH cells compared to control upon cisplatin treatment (Fig. 1b).

To measure the accumulation of cisplatin-induced DNA adducts in cellular DNA upon the treatment, we used dot blot analysis with an antibody specific to cisplatin-modified DNA. HeLa S3 WT and NEIL3-FH cells were exposed overnight to 15 µg/mL cisplatin, then collected and lysed to isolate genomic DNA. The dot blot assay of cellular DNA from WT cells exposed to cisplatin overnight showed an intense signal, indicating accumulation of a high level of cisplatin-modified DNA, whereas NEIL3-FH cells exhibit a much lower signal, as compared to WT (Fig. 1c), suggesting enhanced removal of the cisplatin-induced DNA damage. In agreement with the results of the apoptosis assay, the dot blot results suggest that the overexpression of the exogenous NEIL3 protein increases cell resistance to cisplatin by preventing the accumulation of cisplatin–DNA adducts.

Next, we used the "loss-of-function" approach to corroborate further the role of NEIL3 in the protection from cisplatin-induced apoptosis and DNA damage. The *NEIL3* gene knockout HeLa S3 cell lines were constructed using the CRISPR/Cas9 technique. Different combinations of two gRNAs that target the first exon of the *NEIL3* gene were used to avoid similar off-target effects. Disruption of the *NEIL3* gene and the lack of NEIL3 mRNA expression were confirmed by genomic DNA sequencing and RT-qPCR, respectively (Figs. S1a and 1d).

In agreement with data obtained with HeLa S3 NEIL3-FH cells, NEIL3 knockout (NEIL3^-/-^) cells exhibit increased levels of apoptosis (Fig. 1e) and higher accumulation of cisplatin–DNA adducts as compared to that of control WT cells (Fig. 1f). To meet the required standards, we validated our results obtained with HeLa S3 cells on the NEIL3^-/-^ knockout U2OS cell lines (Fig. S1b–d) and on the A549 cell line with siRNA-induced downregulation of NEIL3 (Fig. S1e–g). Taken together these results suggest that NEIL3 protects cells from cisplatin–DNA adduct-induced apoptosis through their removal.

### The activity of 26S proteasome is essential for the NEIL3-mediated cisplatin resistance

**Figure 2:**
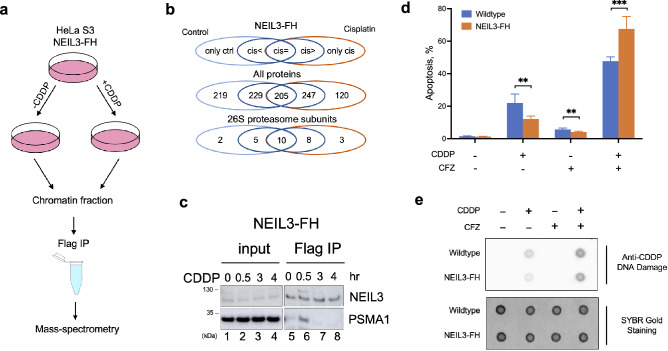
26S proteasome plays a role in the NEIL3-mediated removal of cisplatin–DNA adducts. (**a**) Schematic outline of the mass-spectrometry analysis of Flag IP samples performed before and after 30 min treatment of HeLa S3 NEIL3-FH cells with 15 µg/mL cisplatin. (**b**) Venn diagrams representing the results of mass-spectrometry: “only ctrl” denotes proteins found in control non-treated cells; “cis < ” and “cis > ” indicate proteins that increase or decrease, respectively, after cisplatin treatment; “cis = ” refers to proteins that do not change after the treatment; “only cis” designates proteins that are detected only after the treatment. (**c**) Western blot of Flag IP samples purified from chromatin fractions of NEIL3-FH cells treated with cisplatin (CDDP, 15 µg/mL) for the indicated period of time. Note that the membrane was cropped to remove irrelevant parts. (**d**) Measurement of apoptosis by flow cytometry of Annexin V and Propidium Iodide (PI) stained cells pretreated with 0.5 µM CFZ followed by overnight incubation with different doses of CDDP. Data shown are the mean ± SD (n = 3). **p <0.01; ***p <0.001. (**e**) Dot blot with an antibody recognizing cisplatin-modified DNA to measure the accumulation of cisplatin–DNA adducts after the pretreatment with 0.5 µM CFZ followed by overnight exposure to CDDP (15 µg/mL). SYBR Gold staining was used as a loading control. See Materials and Methods for additional details.

An extended non-catalytic C-terminal domain (CTD) is a particular structural feature of NEIL3, absent in the paralogous NEIL1 and NEIL2 proteins (Fig. S2a). The CTD of NEIL3 contains additional zinc finger motifs responsible for DNA–protein and protein–protein interactions (Fig. S2a). The Ran Binding Protein-type 2 zinc finger (RanBP ZF) of NEIL3 was shown to interact with ubiquitylated CMG (Cdc45-MCM-GINS complex is the eukaryotic replicative helicase that unwinds duplex DNA at replication forks)^9^, whereas two tandem glycine-arginine-phenylalanine type zinc finger (GRFs) are responsible for the interaction with ssDNA and fork-like DNA structures^9–11^. Thus, the CTD of NEIL3 could bind to ssDNA and recruit various DNA repair proteins and initiate the formation of specific multi-protein complexes at the stalled replication fork to facilitate the removal of complex DNA lesions such as ICLs. The existence of this domain suggests that the NEIL3-mediated DNA repair may involve additional interacting proteins. We next searched for the protein partners of NEIL3 that could be involved in the repair of cisplatin-induced DNA damage. HeLa S3 NEIL3-FH cells were treated with cisplatin, followed by fractionation of cell extracts into soluble and chromatin fractions and isolation of NEIL3 complexes from each fraction by Flag immunoprecipitation (Flag IP) (Fig. 2a). Mass-spectrometry of samples isolated from the chromatin fraction revealed the presence of more than 20 subunits of the 26S proteasome; their abundance increased after 30 min of cisplatin treatment (Fig. 2b). The interaction between NEIL3 and 26S proteasome was further confirmed by western blot analysis using PSMA1 antibody, which recognizes the α6 core subunit of 26S proteasome. The western blot revealed a transient stabilization of the NEIL3/26S proteasome complex after 30 min exposure (Fig. 2c, lane 6). However, after 3–4 h of cisplatin treatment, we did not detect NEIL3/proteasome interaction (Fig. 2c, lanes 7–8). The degradation of NEIL3 could potentially explain the loss of this interaction. However, the amounts of NEIL3-Flag/HA purified by Flag IP were approximately the same at different time points after cisplatin treatment (Fig. 2c, lanes 5–8), suggesting that NEIL3 is not degraded at least during the first hours. Therefore, we may suggest that loss of NEIL3/proteasome interactions could also be explained by the timely disappearance of a third protein mediating this interaction.

To examine whether proteasome plays a role in the NEIL3-mediated protection from cisplatin-induced apoptosis, we exposed HeLa S3 cells to either carfilzomib (CFZ, an irreversible proteasome inhibitor), or cisplatin, or both drugs together. Co-treatment of NEIL3-FH cells with cisplatin and CFZ led to the accumulation of high levels of cisplatin–DNA adducts and abolished the protective effect of NEIL3 (Fig. 2d,e). Taken together, these data suggest that the activity of 26S proteasome is essential for the NEIL3-dependent repair of the cisplatin–DNA adducts and protection from apoptosis.

### WRNIP1/NEIL3 interaction is regulated via NEIL3-mediated proteasomal degradation during the repair of cisplatin-induced DNA damage

**Figure 3:**
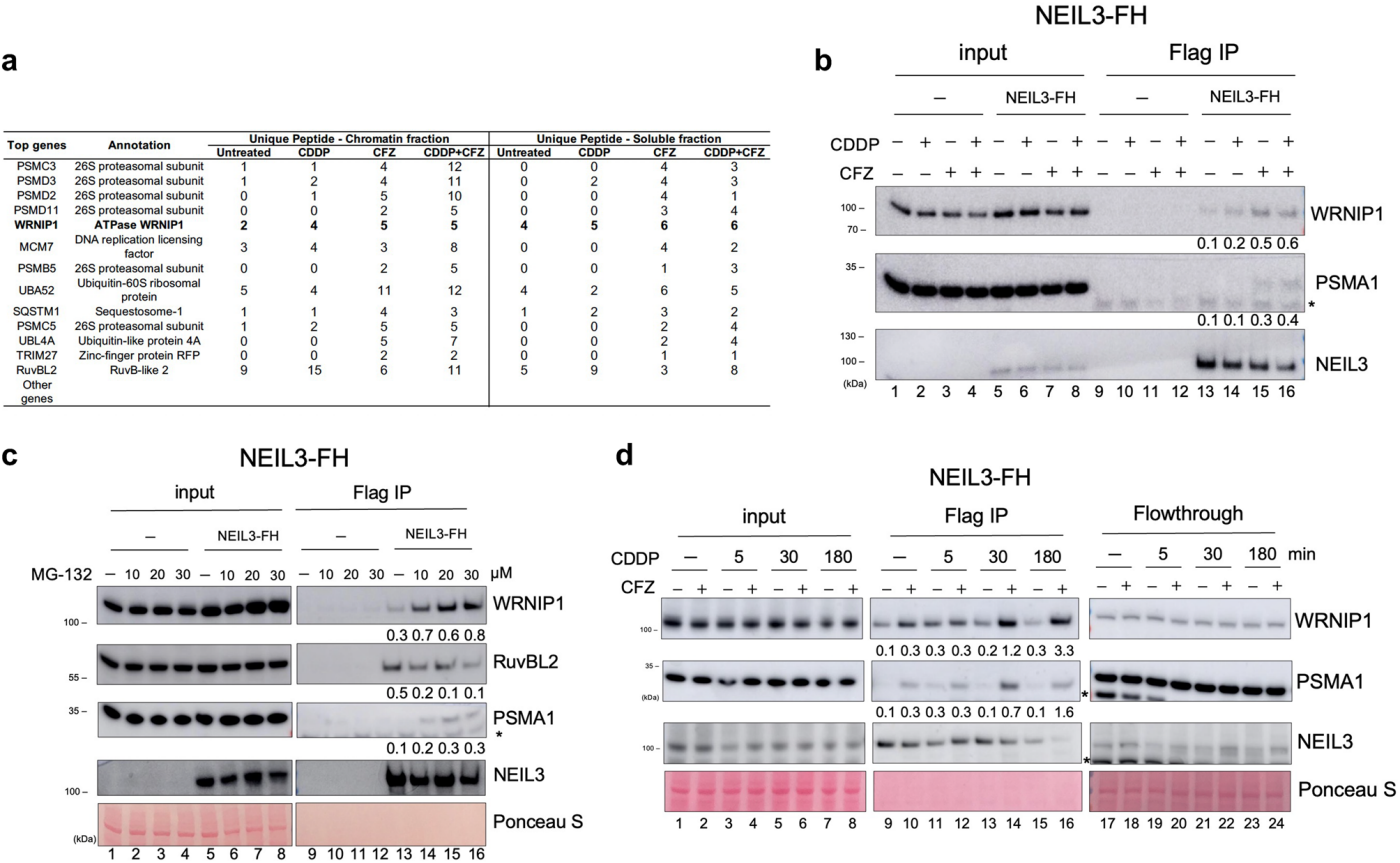
WRNIP1 interaction with the 26S proteasome is stabilized by the proteasome inhibitors. (**a**) Proteins identified by mass spectrometry in the NEIL3-Flag/HA protein complexes analyzed before and after 3 h treatment with either 15 µg/mL CDDP or 0.5 μM CFZ, or a combination of both. (**b**) Western blot of Flag IP samples purified from NEIL3-FH cells treated with CDDP, CFZ, or both drugs. (**c**) Western blot of Flag IP samples purified from NEIL3-FH cells treated with different doses of MG-132, a reversible proteasome inhibitor. (**d**) Western blot of Flag IP samples purified from NEIL3-FH cells pre-treated with 0.5 μM CFZ followed by 15 µg/mL CDDP treatment for different time points demonstrating the dynamics of interaction between NEIL3, WRNIP1 and 26S proteasome. Note that membranes were cropped to remove overexposed parts. IP experiments were normalized by Flag/HA tagged protein used as a bait and quantified using ImageJ software. Ratios for each protein are indicated below the given blot. See Materials and Methods for additional details.

Here we hypothesized that NEIL3/proteasome interaction is required for a targeted protein degradation during the repair of stalled replication fork. To identify a potential proteasome substrate in the NEIL3-mediated repair pathway, another mass-spectrometry analysis of protein complexes isolated by Flag IP from NEIL3-FH cells treated with either CFZ, cisplatin, or both drugs was performed. Cells were treated for 3 h because proteasome content in the NEIL3 protein complexes isolated from the chromatin fractions peaks around 30 min, but then decreases dramatically 3 h after the start of cisplatin treatment (Fig. 2c, lanes 7–8). Here, we searched for the proteins whose levels increased after CFZ treatment. As expected, analysis of the spectra and dynamics of peptides confirmed the increased association of proteasome with NEIL3 (Fig. 3a). Several other candidates were detected, of which WRNIP1 has been selected to follow due to its established role in ICL repair^34^. The western blot confirmed the proteomics data by showing the stabilization of WRNIP1/NEIL3 and NEIL3/proteasome interactions in CFZ-treated NEIL3-FH cells (Fig. 3b, lanes 13–16). These results suggest that WRNIP1 may be a third partner in the NEIL3/proteasome complex. A dose-dependent stabilization of WRNIP1 in Flag IP purified NEIL3 complexes from the cells treated with another proteasome inhibitor MG-132 was also observed (Fig. 3c, lanes 13–16). These results suggest that the WRNIP1 protein is a NEIL3-associated specific target of proteasomal degradation. On the other hand, RUVBL2, a protein that plays a role in the repair of psoralen-induced ICLs and has been recently shown to interact with NEIL3^15^, did not show any stabilization after the treatment with either CFZ or MG-132 (Fig. 3a,c lane 13–16). This observation might suggest that the NEIL3-dependent degradation of WRNIP1 is specific for the repair of cisplatin-induced DNA damage, whereas interaction of NEIL3 with RUVBL2 may result in a different outcome.

To determine whether WRNIP1 interacts with NEIL3 and proteasome in transient manner, which can be stabilized by proteasome inhibitors, we performed the Flag IP experiments in extracts of NEIL3-FH cells treated by cisplatin for different times (0–3 h) in the presence or absence of proteasome inhibitor CFZ, which was added to cultures 1 h before cisplatin (Fig. 3d). We detected an increase of WRNIP1 in NEIL3 complexes after a 5 min cisplatin treatment in the absence of CFZ, but it declined slightly at 30 min, and almost disappeared after 3 h treatment. When cells were pretreated with CFZ, the level of WRNIP1 in the NEIL3 complex did not decline. The proteasome in the complex, revealed with PSMA1 (α6 subunit) antibodies, exhibit the same dynamics as WRNIP1 and increased after CFZ treatment. The levels of WRNIP1 did not change in the flow-through of NEIL3 Flag IP, strongly suggesting that time-dependent loss of WRNIP1 from NEIL3 complexes is caused by its proteasomal degradation and not by the dissociation of WRNIP1 from the NEIL3 protein complex (Fig. 3d, lanes 17–24). Noteworthy, treatment with CFZ increased the amount of NEIL3-associated WRNIP1 in the absence of cisplatin treatment (compare lanes 9 and 10), suggesting continuous turnover of NEIL3-bound WRNIP1 by 26S proteasome present in complexes, which is somehow blocked during rapid recognition of cisplatin-induced ICLs. It should be noted that the level of NEIL3 decreased upon extended exposure of cells to cisplatin, but as compared to WRNIP1, it was not dependent on the presence of CFZ (lanes 9–16). This may suggest that variations in the level of NEIL3 upon cisplatin exposure are not dependent on the proteasome. We further hypothesized that the stabilization of NEIL3/WRNIP1 interactions after exposure to cisplatin plays an important role in the rapid recognition of the stalled replication forks at cisplatin-induced ICLs, which enables the initiation of early steps of their repair.

The CTD of NEIL3 might be involved in the specific interactions with WRNIP1. To examine this, we used previously generated HeLa S3 NEIL3^-/-^ cell line and constructed cell lines expressing the FH-tagged: (i) NEIL3-FL protein; (ii) N-terminal truncated NEIL3-CTD protein (lacking catalytic DNA glycosylase domain) and (iii) C-terminal truncated NEIL3-NTD protein (containing only catalytic DNA glycosylase domain and lacking completely CTD). Western blot of the IP Flag samples shows almost the same efficiency of the interaction between WRNIP1 versus NEIL3-FL and NEIL3-CTD, but no interaction is observed between WRNIP1 and NEIL3-NTD, suggesting that CTD, but not NTD domain is involved in the interactions with WRNIP1 (Fig. S2b,c).

### NEIL3-dependent degradation of WRNIP1

**Figure 4:**
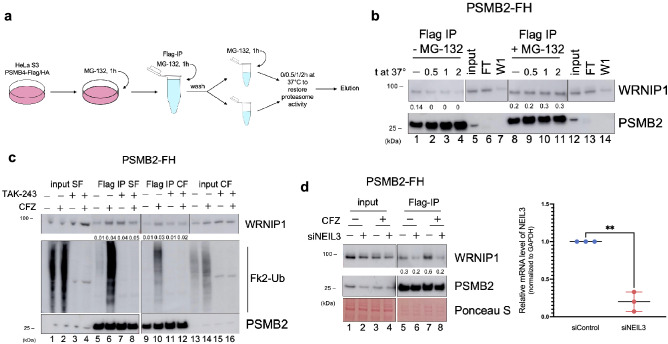
WRNIP1 is degraded in the ubiquitin-proteasome pathway. (**a**) Schematic outline of the experiment shown in panel “b”. (**b**) Western blot of Flag IP samples purified from PSMB2-FH cells treated with MG-132 as depicted in panel “a”. (**c**) Western blot of Flag IP samples purified from HeLa S3 PSMB2-FH cells treated with broad ubiquitylation inhibitor TAK-243 and carfilzomib. (**d**) Effect of NEIL3 knockdown on WRNIP1 degradation. Left, Western blot of Flag IP samples purified from PSMB2-FH cells treated with cisplatin, CFZ, and NEIL3 siRNA; Right, confirmation of NEIL3 knockdown by RT-qPCR. Data shown are the mean ± SD (n = 3). **p <0.01. Note that the membranes were cropped to remove overexposed parts. CF, chromatin fraction; SF, soluble fraction that contains cytoplasmic and nuclear proteins; FT, flow-through; W1, first wash of the beads. IP experiments were normalized by Flag/HA tagged protein used as a bait and quantified using ImageJ software. Ratios for each protein are indicated below the given blot. See Materials and Methods for additional details.

To further investigate whether WRNIP1 in the complex with NEIL3 and 26S proteasome is degraded, we designed a “degradation on the beads” experiment. We used HeLa S3 cells expressing the PSMB2, a β4 subunit of the 20S proteolytic core of the proteasome tagged with Flag/HA epitopes at the C-terminus. We treated PSMB2-FH cells with a reversible proteasome inhibitor MG-132 for an hour, followed by cell collection and Flag IP in the presence of MG-132 to keep the isolated proteasome inhibited. Before the elution, beads were washed and divided into two parts: with and without MG-132. Then they were incubated at 37 °C in the presence of ATP to maintain the integrity and activity of the 26S proteasome and to allow protein degradation (Fig. 4a). Protein complexes were then eluted with the Flag peptide and analyzed by western blot. In the samples in which MG-132 was removed, we detected the gradual disappearance of WRNIP1 (Fig. 4b, lanes 1–4), whereas the amount of WRNIP1 did not change when the inhibitor was present (Fig. 4b, lanes 8–11). In addition, we observed the stabilization of WRNIP1 and the appearance of high molecular weight smear characteristic of ubiquitin chains in Flag IP samples from CFZ-treated PSMB2-FH cells (Fig. S2d, lanes 5–8, 13–16). Thus, proteasome-bound WRNIP1 is degraded by the ubiquitin–proteasome pathway (UPP).

Most substrates of the 26S proteasome are poly-ubiquitylated before degradation and bind to the proteasome via ubiquitin chains. To determine whether WRNIP1 binds to proteasome via the polyubiquitin chain, we used TAK-243, a specific inhibitor of the E1 ubiquitin-activating enzyme^36^, which completely blocks ubiquitylation. Flag IP samples from HeLa S3 PSMB2-FH cells treated with TAK-243 were analyzed by Western blot using FK2-Ub antibody that recognizes the polyubiquitin chains (Fig. 4c). Surprisingly, the interaction of WRNIP1 with proteasome was not blocked by TAK-243 treatment (lanes 7 and 11). Furthermore, the levels of proteasome-bound WRNIP1 were significantly stabilized by TAK-243 even in the absence of CFZ (lanes 5–8, 9–12), thus indicating that ubiquitylation, although not required for binding to the proteasome, is necessary for WRNIP1 degradation.

In the previous experiment, we detected CFZ-induced changes in the WRNIP1 levels in NEIL3 complexes but not in whole-cell extracts (Fig. 3b–d), suggesting that WRNIP1 is degraded only upon interaction with NEIL3. To determine whether NEIL3 targets WRNIP1 to the proteasome, we analyzed the effect of NEIL3 knockdown on proteasomal degradation of WRNIP1 in PSMB2-FH cells (Fig. 4d). As expected, CFZ-induced stabilization of the interaction of WRNIP1 with proteasome was not observed in cells treated with NEIL3 siRNA (lanes 6 and 8). These results strongly suggest that NEIL3 is required for WRNIP1 binding to the proteasome, which eventually results in WRNIP1 degradation.

## Discussion

In addition to its well-established role in the classic BER pathway for oxidative DNA base damage, NEIL3 DNA glycosylase plays a major role in repairing the psoralen-induced DNA cross-links in human cells and is the first-line defense against ICLs due to its rapid recruitment to the sites of DNA damage^15^. In the present work, as shown in Fig. 1, we confirm the previously published observations that NEIL3 knock-out cells are sensitive to cisplatin^15,25^. We have previously demonstrated that NEIL3 can unhook psoralen-induced ICLs in vitro via its DNA glycosylase activity^16–18^. It has been shown that despite the rapid recruitment of NEIL3 to cisplatin-induced ICL in Xenopus egg extracts, this DNA glycosylase cannot excise cisplatin–DNA adduct ^12,19^. In the present study, we demonstrate that nevertheless NEIL3 plays a role in the repair of cisplatin-induced DNA adducts in human cells, possibly as scaffold protein, which helps targeting the specific protein substrates to proteasomal degradation in cisplatin dependent manner. Together with the observations in literature showing that loss of NEIL3 markedly increases replication-associated DSB formation, and that NEIL3 overexpression is associated with genomic alterations and poor prognosis in cancer^20,21^, our findings further suggest that NEIL3 can contribute to cisplatin resistance.

Unlike other members of the Fpg/Nei family, NEIL3 DNA glycosylase has an extended C-terminal domain which participates in the protein–protein and DNA–protein interactions^12–14^. To get insight into the molecular mechanism of NEIL3-mediated cisplatin-induced ICLs repair, we searched for the protein partners that bind to NEIL3 partners in a cisplatin-dependent fashion and identified 26S proteasome as a cisplatin-dependent interacting partner (Fig. 2). Importantly, CFZ, a proteasome inhibitor, abolishes the NEIL3-mediated protection from cisplatin-induced apoptosis and increases the level of DNA cross-links (Fig. 2) suggesting that the proteasomal degradation is required to repair cisplatin-induced DNA adducts.

It was shown that targeted degradation of the specific proteins is required for the timely completion of DNA repair. For example, the ubiquitin-independent degradation of acetylated histones by 20S proteasome-PA200 complex during repair of double-strand breaks and UV-induced DNA damage^36,37^. There are several examples of the ubiquitin-dependent degradation in DNA repair pathways. Degradation of MDC1 appears to be a key step in the assembly of BRCA1 foci during DSB repair^38^. Timely removal of RPA1 and RAD51 at DNA damage sites facilitates homologous recombination^39^. The large subunit of stalled RNA polymerase II is degraded upon DNA damage to clear transcriptionally active genes from highly toxic, permanently arrested RNAPII elongation complexes^40^. Timely destruction of DDB2 is important for nucleotide excision repair^41^. Another example is the CSA-dependent degradation of CSB by the 26S proteasome during transcription-coupled nucleotide excision repair^33^. In patients with Cockayne syndrome (CS), a rare genetic disorder characterized by progressive neurological degeneration and premature aging, inability to remove the CSB protein due to mutation in CSA and CSB genes, leads to a deficiency in the transcription recovery after DNA damage^41^.

In this study, we identify WRNIP1 as a proteasome substrate degraded during ICL repair. Stabilization of WRNIP1 immediately after damage and its subsequent timely destruction resembles timely destruction of cyclins and inhibitors of cyclin-dependent kinases during the cell cycle. The degradation of these proteins is regulated by phosphorylation-dependent ubiquitylation^42^. Similarly, WRNIP1 ubiquitylation can be regulated by DNA-damaged induced phosphorylation of a yet-to-be-identified ubiquitin ligase. Alternatively, it is possible that the turnover of proteasome-bound WRNIP1 is blocked by DNA-damage-induced activation of deubiquitylating enzymes^43^. Finally, spatially restricted temporally inhibition of proteasome cannot be ruled out. The proteasome is present in the specific region of chromatin, including sites of DNA damage^44^, but the mechanism of proteasome recruitment to the sites of DNA damage is unknown. Although it is possible that proteasome simply binds to proteins ubiquitylated on chromatin, our data suggest that its recruitment to cisplatin-induced DNA lesions is NEIL3-dependent.

**Figure 5:**
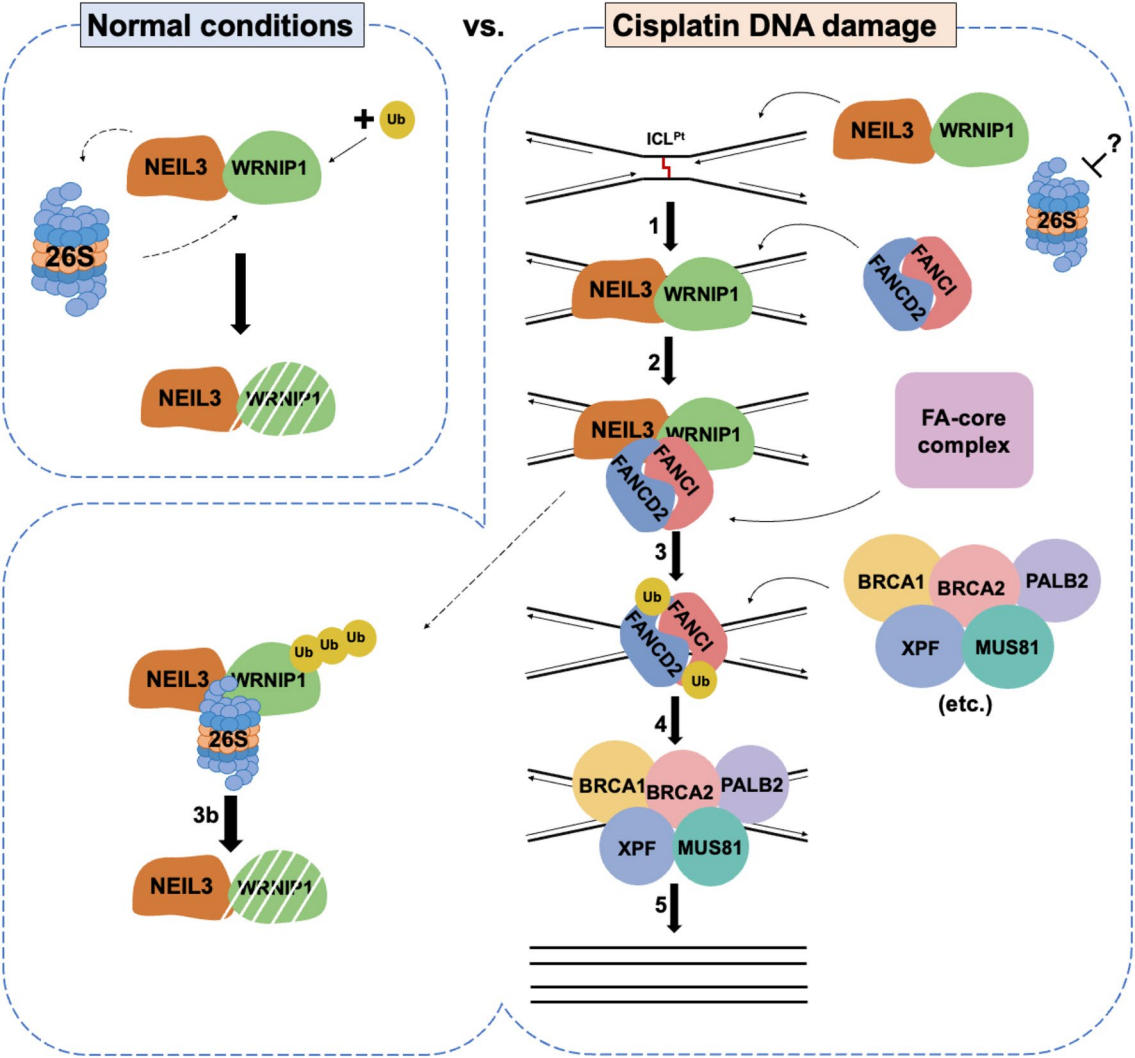
A model of the NEIL3-dependent proteasomal degradation of the WRNIP1 protein upon cisplatin-induced ICLs.

Here, to integrate our findings with the published literature we propose a putative model of the NEIL3-mediated ICL repair (Fig. 5). WRNIP1 as an ICL repair protein is constantly degraded in unstressed cells (Fig. 5, left). This proteasomal degradation of WRNIP1 occurs in NEIL3-dependent manner (Fig. 4d). However, upon the generation of cisplatin-induced ICL, the NEIL3-dependent degradation of WRNIP1 is blocked and both proteins are recruited to the ICL-stalled replication fork (Fig. 5, step 1). WRNIP1 then recruits FANCD2/FANCI dimer as shown by Socha and colleagues^34^ (Fig. 5, step 2), FANCD2 and FANCI are mono-ubiquitylated by the Fanconi core complex (Fig. 5, step 3), which enable the recruitment of repair module proteins (BRCA1, BRCA2, XPF-ERCC1 and etc.; Fig. 5, step 4). WRNIP1 is ubiquitylated and degraded (Fig. 5, step 3b), perhaps with other early-stage repair proteins, facilitating the high-fidelity repair of complex DNA lesion such as ICL (Fig. 5, step 5). Thus, the interplay between WRNIP1 and NEIL3 channels the repair of the cisplatin induced ICLs toward the FA pathway to ensure safe and high-fidelity DNA repair.

## Materials and methods

### Cell lines and culture conditions

HeLa S3 and U2OS cells were purchased from the American Type Culture Collection (ATCC, Rockville, MD) and grown in Dulbecco’s modified Eagle medium (DMEM) supplemented with 10% (v/v) fetal bovine serum (ThermoFisher Scientific) and 1% penicillin/streptomycin (ThermoFisher Scientific) in a 5% CO_2_ incubator at 37 °C. The non-small cell lung cancer (NSCLC) A549 cell line was kindly provided by Dr. Maria Castedo (INSERM U1138, Paris, France)^45^ and was maintained in DMEM Nutrient Mixture F-12 (DMEM/F-12) supplemented with 10% FBS and 1% penicillin–streptomycin in a 5% CO_2_ incubator at 37 °C.

### Generation of NEIL3-Flag/HA cell lines

To obtain a stable HeLa S3 NEIL3-Flag/HA overexpressing cell line, human NEIL3 cDNA was subcloned into the pOZ-C retroviral expression vector by restriction enzyme digestion with XhoI and NotI. To confirm proper integration and lack of mutation in the NEIL3 gene, we then sequenced the pOZ-C-NEIL3-Flag/HA plasmid around the insertion site. To encapsulate the construct for retroviral transduction, Phoenix-A cells were seeded at the density of 120,000 cells per one well at a 6-well plate and transfected with the construct using Lipofectamine 2000. At 48 and 72 h post-transfection, the supernatant containing retrovirus was collected and filtered with a 0.22 mm filter and added to HeLa S3 cells seeded in a 6-well plate at 70% confluency. The cells were sorted 48 h later using DynaBeads™ (Invitrogen) in an Isolation buffer (Ca^2+^ and Mg^2+^-free PBS supplemented with 0.1% BSA and 2 mM EDTA, pH7.4). NEIL3-Flag/HA overexpression was validated by western blot.

### Generation of NEIL3^-/-^ cell lines

HeLa S3 *NEIL3*^*-/-*^ cell lines were generated using the CRISPR-Cas9 system. Oligonucleotides encoding different guide RNAs (#1-5′-GCGCAGCGTTGAGTTGCACAG-3′, #2-5′-CGGTTGTGGTCTCCCCGCAG-3′, #3-5′-CTCATTTAGGAGTCGACTGC-3′) targeting the *NEIL3* gene were cloned into the PX458 vector, containing Cas9 and a GFP selection marker. HeLa S3 cells were seeded at 60% confluence in 6-well plate dishes 24 h before transfection. The gRNA-PX458 construct was transfected using the JetOPTIMUS (Polyplus). The selection of cells based on GFP fluorescence was done 48 h after the transfection on the Aria II Flow Cytometer (BD Biosciences). Clones were collected after 3 weeks and the disruption of NEIL3 was identified by sequencing and RT-qPCR.

### siRNA transfection

siRNA oligonucleotides were transfected into cells using INTERFERin transfection agent (Polyplus) according to the manufacturer’s instructions. The sequences of the siRNAs used in this study: siLac (control): 5′-CGUCGACGGAAUACUUCGA-3′; siNEIL3: 5′-GGGUGGAUCAUGUUAUGGA-3′.

### Reverse transcription-quantitative PCR (RT-qPCR)

RNA was isolated using the NucleoSpin RNA kit (Macherey–Nagel, USA) and used for the generation of cDNA by reverse transcription using the High-Capacity cDNA Reverse Transcription Kit with RNase Inhibitor (Applied Biosystems). SYBR Select Master Mix (Life Technologies) and the following primers were used for qPCR: NEIL3 #1: forward, 5′-AGTGGTCTCCACCCAGCTGTTA-3′; reverse, 5′-AGAGCAAGTCCTGCTTTACGGC-3′; NEIL3 #2: forward, 5′-GGTCTCCACCCAGCTGTTAAAG-3′; reverse, 5′-CACGTATCATTTTCATGAGGTGATG-3′; GAPDH: forward, 5′-CTGCACCACCAACTGCTTAG-3′; reverse, 5′-AGGTCCACCACTGACACGTT-3′.

### Apoptosis assay

Cells were seeded in 12-well plates in a quantity of 100,000 cells per well. The next day, different doses of cisplatin (Mylan, France) were added, followed by overnight incubation at 37 °C with 5% CO_2_. For the detection of apoptosis induction, cells were collected and incubated in 300 µL binding buffer (0,1 M Hepes (pH 7.4); 1,4 M NaCl; 25 mM CaCl_2_) with 3 µL Annexin V-FITC and propidium iodide (PI, 2,5 µg/mL). BD Accuri C6 flow cytometer was used to detect apoptosis induction.

### Cell fractionation

Cells were collected by scraping and washed with phosphate-buffered saline (PBS) followed by resuspension in 1 × micrococcal nuclease (MNAse) buffer (200 mM Tris HCl, 25 mM CaCl_2_, 50 mM NaCl) supplemented with 1 × protease inhibitor cocktail (PIC), and then subjected to lysis by freezing them at − 80 °C. Lysed cells were centrifuged at 16,000 × g and the supernatant was collected as a ‘soluble fraction’. To isolate chromatin-bound proteins, the cell pellet was resuspended in MNAse buffer and treated with MNAse (Sigma) for 10 min on ice and centrifuged at 16,000 × g for 5 min. The remaining pellet was subjected to extraction with a 10 × TBS buffer and washed with 1 × TBS buffer. The supernatant from these three steps was combined and considered as a ‘chromatin fraction’.

### Dot blot assay

To analyze the accumulation of cisplatin-induced DNA damages, cells were seeded in 10 cm dishes, and at 70% of confluence, cells were treated with cisplatin and/or carfilzomib for indicated times followed by chromatin fraction isolation described above. DNA was isolated using phenol–chloroform extraction method. DNA concentrations were measured by a Biospec-Nano spectrophotometer. 300 ng of DNA in 3 µl of water was loaded in a Hybond filter and UV cross-linked on UV Stratalinker 2400™ (Stratagene). Then membranes were incubated in 1 × TBST with 1:10,000 SYBR Gold (Life Technologies) for 30 min and washed with 20% ethanol for 20 min. Typhoon FLA 9500 was used to detect DNA signal with SYBR Gold fluorescent dye for loading control. Followed this, membranes were incubated for 1 h in 10% milk for blocking and incubated overnight in antibodies against cisplatin–DNA adducts (Abcam, dilution 1:1000). Then the membrane was washed in 1 × TBS-T three times for 5 min, and cisplatin-induced lesions were detected using anti-rat antibodies.

### Flag immunoprecipitation (flag IP)

HeLa S3 cells were seeded in 15 cm dishes to reach 80% confluence the next day and were treated with different drugs for the indicated times. Cells were collected by scraping and washed with phosphate-buffered saline (PBS) followed by spinning at 500 × g for 2 min. Cell fractions were isolated using the protocol described above and normalized in concentration using the Bradford assay followed by incubation with 50 μL Flag agarose beads for 1 h at 4 °C. Then the beads were washed three times with 1 × TBS supplemented with PIC. Protein complexes were eluted from beads using 50 μL of 0.5 mg/mL Flag peptides.

### Immunoblotting and antibodies

For the analysis of whole-cell extracts, cells were collected by scraping from a confluent 6-well plate and washed with PBS. Cell pellets were lysed in a cold RIPA buffer (ThermoFisher Scientific) supplemented with PIC. Samples were normalized by Bradford assay and loaded into a 4–12% Bis–Tris gel. The membranes were blocked in 5% dry non-fat milk in TBS-T for 1 h and then incubated overnight at 4℃ with the following antibodies: anti-HA (Sigma, H3663-100), anti-NEIL3 (Proteintech, 11621-1-AP), anti-WRNIP1 (Santa-Cruz, sc376438), anti-αTubulin (Sigma, T9026), anti-PSMA1 (Mab 2-17, isolated and purified from hybridoma kindly provided by Dr. Keiji Tanaka), anti-RuvBL2 (Proteintech, 10195-1-AP), Fk2-Ub (Merck, 04-263). Proteins were detected using a chemiluminescence system with a horseradish peroxidase-conjugated secondary antibody.

### “Degradation on the beads” protein degradation assay

HeLa S3 PSMB2-FH cells were seeded in 15 cm dishes to reach 80% confluence the next day and were treated with 2 μM MG-132 for 1 h. Cells were collected by scraping and washed with phosphate-buffered saline (PBS) followed Flag IP in the presence of MG-132 to keep 26S proteasome inhibited. Just before the elution, anti-Flag agarose beads with PSMB2 complexes were washed with 1 × TBS supplemented with PIC and divided into two parts—2 μM MG-132 was added back to only one part. We then incubated beads at 37 °C in a special buffer (100 mM Tris (pH7.5), 100 mM KCl, 5 mM MgCl_2_, 1 mM DTT, 1 mM ATP) to restore the activity of a proteasome for indicated times and eluted protein complexes from beads using Flag peptides.

### Mass spectrometry

Wild type and NEIL3-FH HeLa S3 cells were seeded in a 15 cm dish in a quantity of 12 million cells. The next day, cells were treated with 15 µg/mL cisplatin (Mylan, France) for the indicated time periods. For certain samples, 0.5 μM carfilzomib was added 1 h before and kept during cisplatin treatment. NEIL3-Flag/HA and its binding partners were isolated by Flag Immunoprecipitation. The samples were loaded into a 10% Tris–Acetate gel and let migrate for a few centimeters before the bands were cut from the gel and sent to mass spectrometry. The analysis of NEIL3 protein complexes was performed at the Taplin Mass Spectrometry Facility, Harvard Medical School.

### Data analysis

All values shown on the graphs indicate means ± S.D. of several biological replicates, the exact number of which (n) is indicated in the caption. Student’s unpaired *t*-test was applied for two-group comparisons. The asterisks in the graphs correspond to *p*-value ≤ 0.05 for an asterisk (*), *p*-value ≤ 0.01(**), *p*-value ≤ 0.001(***). GraphPad Prism was used for the graphs and statistics.

## Supplementary Information

Supplementary Information.

## Data Availability

The datasets generated during the current study are available in the ProteomXchange repository, http://proteomecentral.proteomexchange.org/cgi/GetDataset?ID=PXD032245 related to Fig. 2 and http://proteomecentral.proteomexchange.org/cgi/GetDataset?ID=PXD034325 related to Fig. 3. Novel cell lines described in this work are available from authors upon request. Further information and requests for resources and reagents should be directed to and will be fulfilled by Dr. Groisman (regina.groisman@gustaveroussy.fr).
