# Supplementary material for: NEIL3-mediated proteasomal degradation facilitates the repair of cisplatin-induced DNA damage in human cells
Source: Sci Rep. 2023 Mar 30;13:5174. doi: 10.1038/s41598-023-32186-3 (PMC10063580; doi:10.1038/s41598-023-32186-3)
Supplement: Supplementary file 1 — Supplementary Information. [file 41598_2023_32186_MOESM1_ESM.pdf]

NEIL3-mediated proteasomal degradation facilitates the repair of cisplatin-induced DNA damage in human cells

Umit Aliyaskarova[1], Yeldar Baiken[2,3,4], Flore Renaud[1,5], Sophie Couve[1,5], Alexei F. Kisselev[6*], Murat Saparbaev[1*], Regina Groisman[1*]

[1]Team «Mechanisms of DNA repair and carcinogenesis», CNRS UMR 9019, Université Paris-Saclay, Gustave Roussy Cancer Campus, F-94805 Villejuif Cedex, France; [2]National Laboratory Astana, Nazarbayev University, Astana, Kazakhstan; [3]School of Sciences and Humanities, Nazarbayev University, Astana, Kazakhstan; [4]School of Engineering and Digital Sciences, Nazarbayev University, Astana, Kazakhstan; [5]EPHE, PSL University, Paris, France; [6]Department of Drug Discovery and Development, Harrison College of Pharmacy, Auburn University, PRB, 720 S. Donahue Dr., Auburn, AL 36849, USA

*co-corresponding authors

Address correspondence to: R.G. and M.S., CNRS UMR 9019, Université Paris-Saclay, Gustave Roussy Cancer Campus, F-94805 Villejuif Cedex
E-mail: R.G. regina.groisman@gustaveroussy.fr and M.S. murat.saparbaev@gustaveroussy.fr
A.F.K. afk0006@auburn.edu

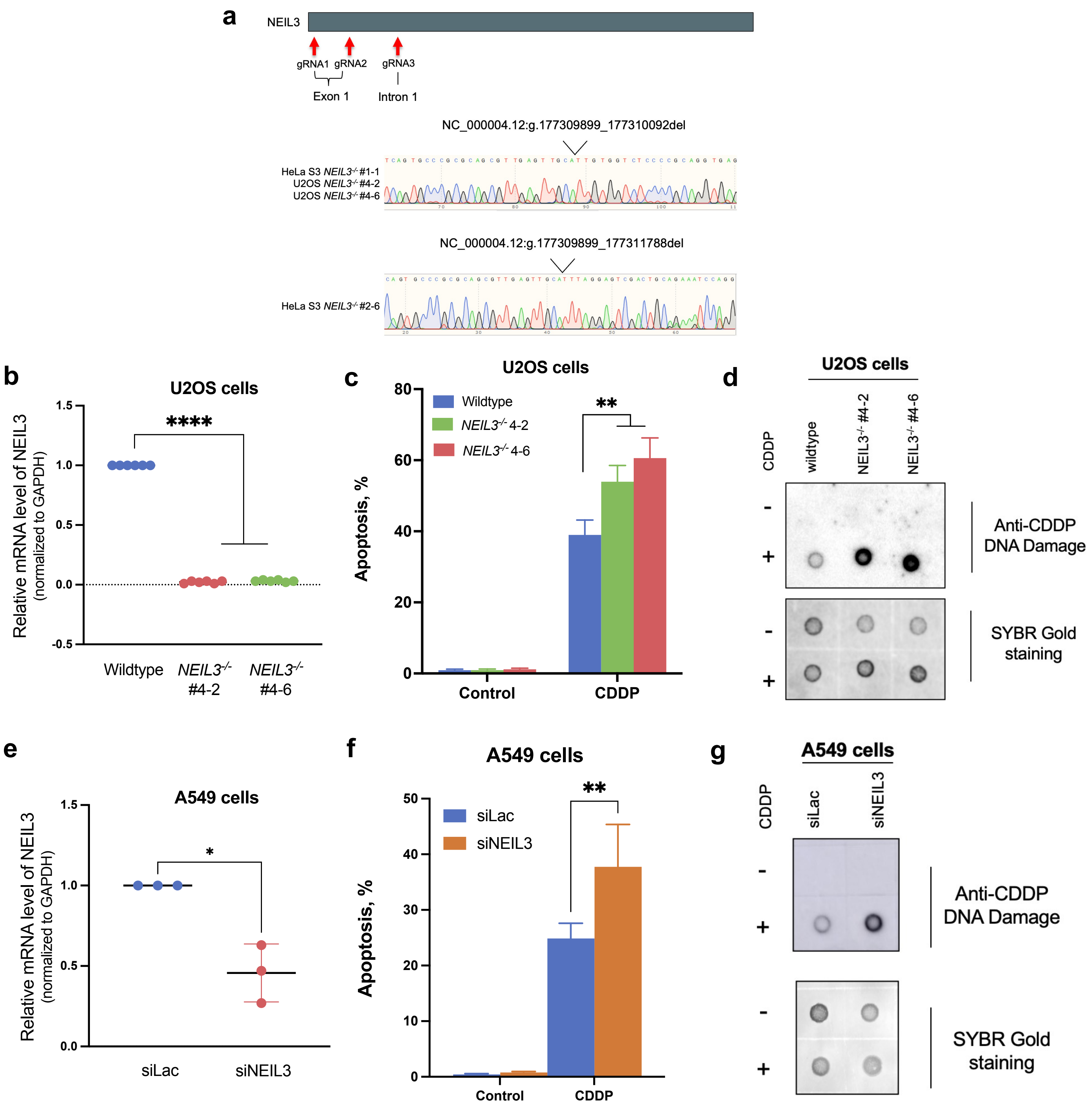

Figure S1. (**a**) Schematic showing CRISPR-Cas9 strategy to create HeLa S3 and U2OS NEIL3$^{-/-}$ cell lines. Two guide RNAs were used to delete a 194 bp region of the first exon and a 1890 bp region in the first exon-intron intersection of *NEIL3* gene. (**b**) Characterization of U2OS cell clones lacking NEIL3, constructed using CRISPR/Cas9 technique. The disruption of the *NEIL3* gene in clones #4-2 and #4-6 was confirmed by RT-qPCR. (**c**) Measurement of apoptosis by flow cytometry of Annexin V and Propidium Iodide (PI) stained cells incubated overnight in the presence of 10 µg/mL of cisplatin (CDDP). Data shown are the mean ± SD (n = 4). **p < 0.01 (**d**) Dot blot with an antibody recognizing cisplatin-modified DNA to measure the accumulation of cisplatin-DNA adducts in cells after overnight exposure to cisplatin (10 µg/mL). SYBR Gold was used as a loading control. (**e**) A549 cells were transfected with siRNA targeting NEIL3. After 48 hours, cells were used for the apoptosis and dot blot assays. NEIL3 knockdown was verified by RT-qPCR. (**f, g**) Same as in "c" and "d" panels except that A549 cells were used.

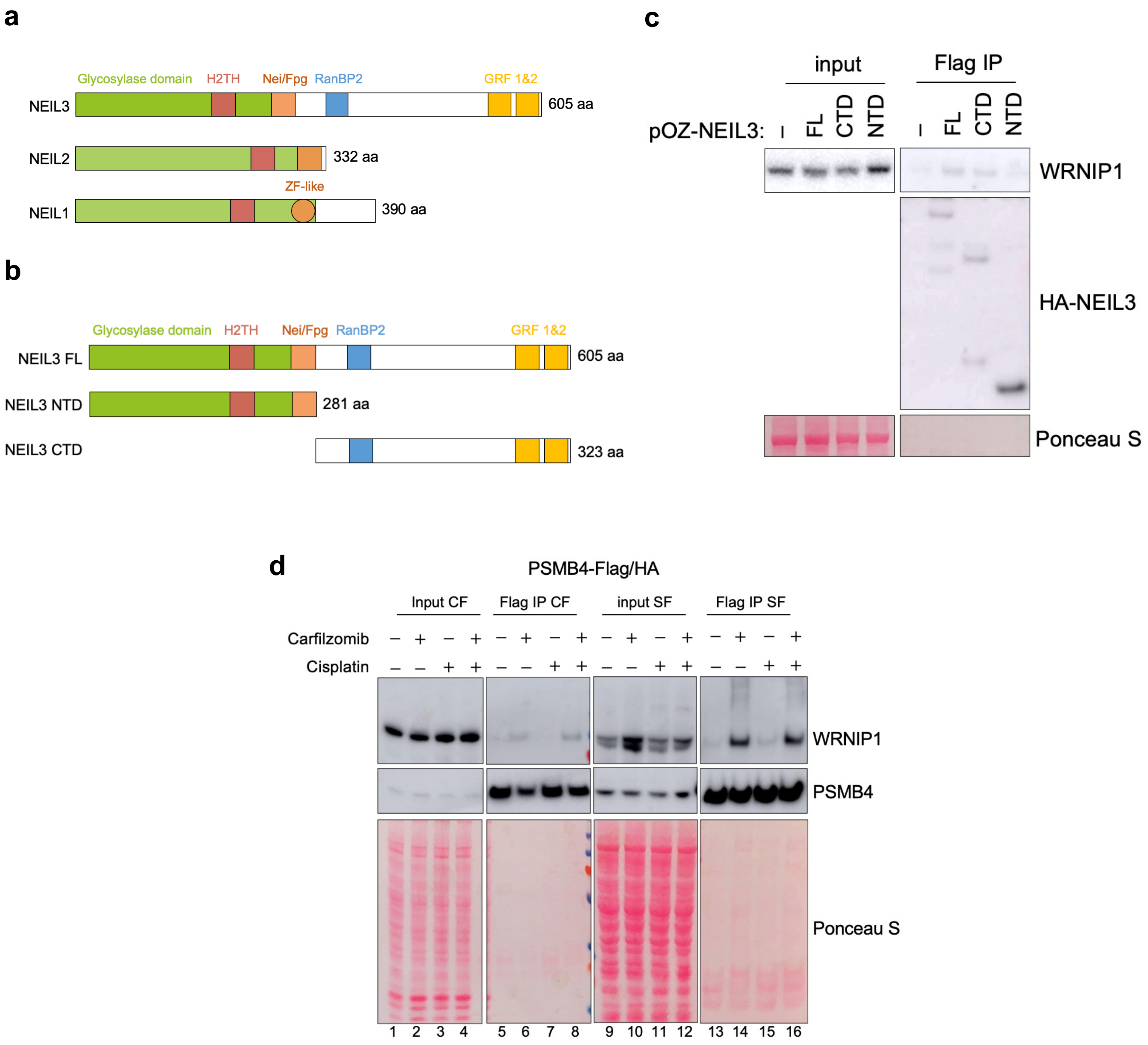

Figure S2. (a) Schematic of the domains of human NEIL3, NEIL2 and NEIL1 proteins that consist of DNA glycosylase domain (green), helix-2-turns-helix (H2TH, red) motif, Nei/Fpg zinc finger (Nei/Fpg, orange), ZF (zinc finger)-like domain (orange round), Ran Binding Protein-type 2 ZF (RanBP2, blue), and two glycine-argininephenylalanine ZF (GRF, yellow) domains; **(b)** Schematic diagram of the full-length NEIL3 protein (NEIL3 FL) domain structures with functional motifs. NEIL3 NTD and NEIL3 CTD denote N-terminal and C-terminal domains of NEIL3, respectively. **(c)** Western blot of Flag IP from HeLa S3 NEIL3$^{-/-}$ cells that exogenously express the full-length (FL), C-terminal (CTD) and N-terminal (NTD) NEIL3 proteins. (Membranes were cropped to remove overexposed parts). **(d)** Western blot of Flag IP samples from HeLa S3 PSMB4 cells treated with cisplatin, and carfilzomib.

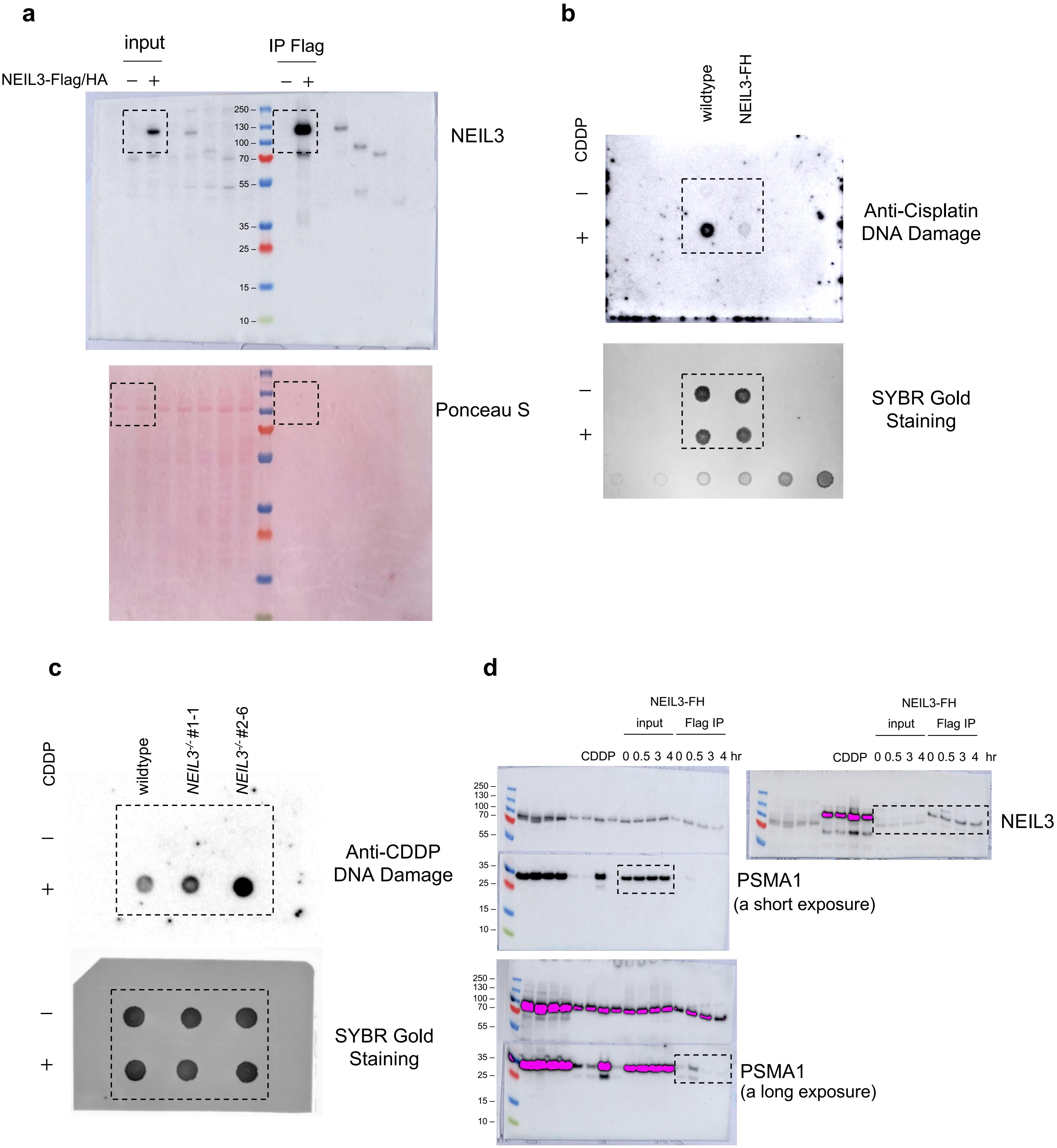

Figure S3. Full scan of the uncropped blots for Figures 1 and 2. (**a**) Original full scan blot used for Fig. 1a. Ponceau S staining was used as a loading control. (**b, c**) Original full scan blots for Fig. 1c, f. SYBR Gold was used as a loading control. Please note that in panel "b" different concentrations of control DNA were loaded to show the sensitivity of SYBR Gold staining. (**d**) Original full scan blots for Fig. 2c. Membrane was cut at ~40kDa and incubated with different antibodies. The upper part of the membrane with PSMA1 was not used in the figure.

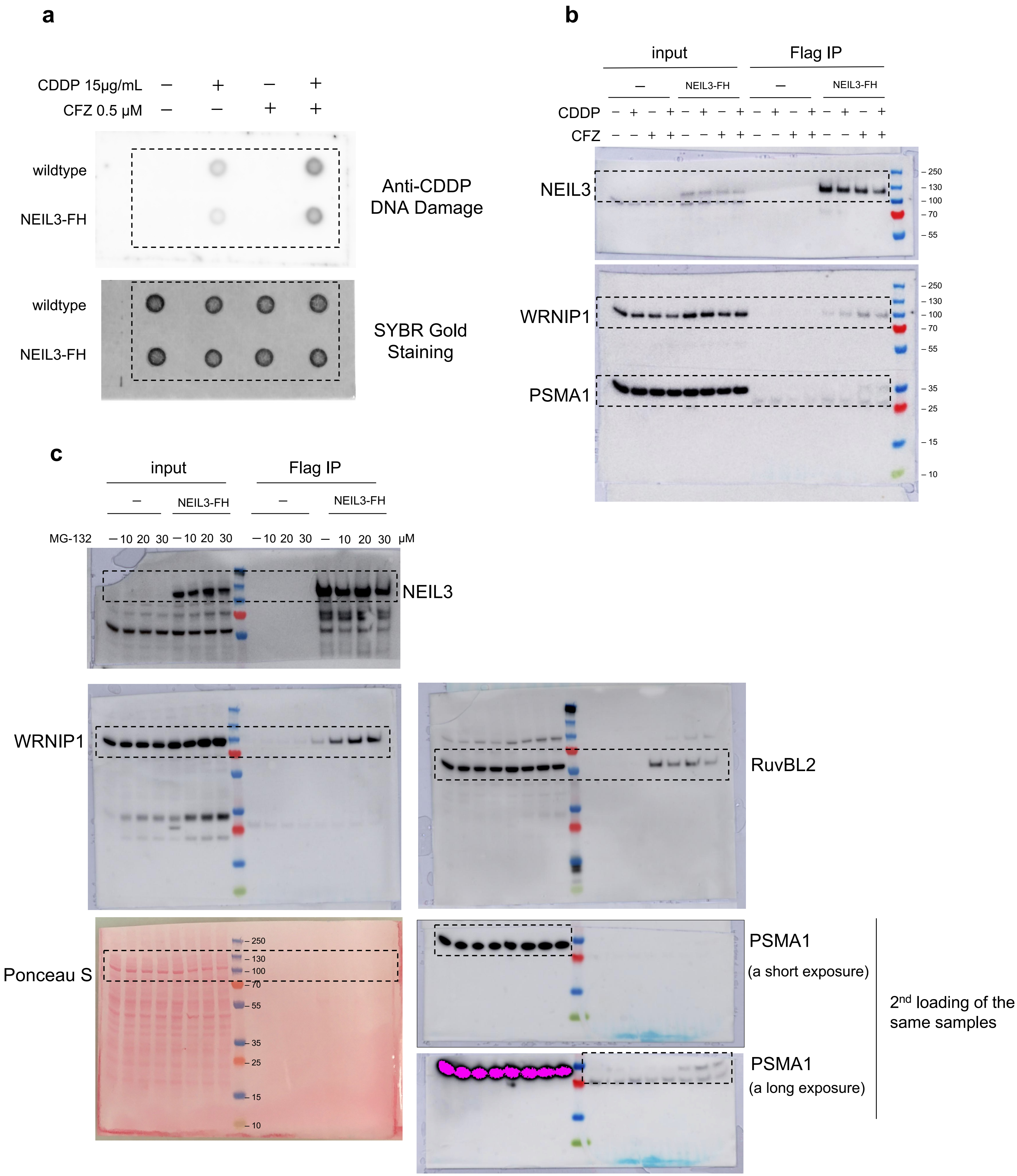

Figure S4. Full scan of the uncropped blots for Figures 2 and 3. (**a**) Original full scan blot for Fig. 2d. SYBR Gold staining was used as a loading control. (**b**) Original full scan blots for Fig. 3b. Membrane was cut at ~40kDa and incubated with different antibodies. (**c**) Original full scan blots for Fig. 3c. Please note that stripping and reprobing of the Western blotting membrane with anti-PSMA1 was used for the same set of samples.

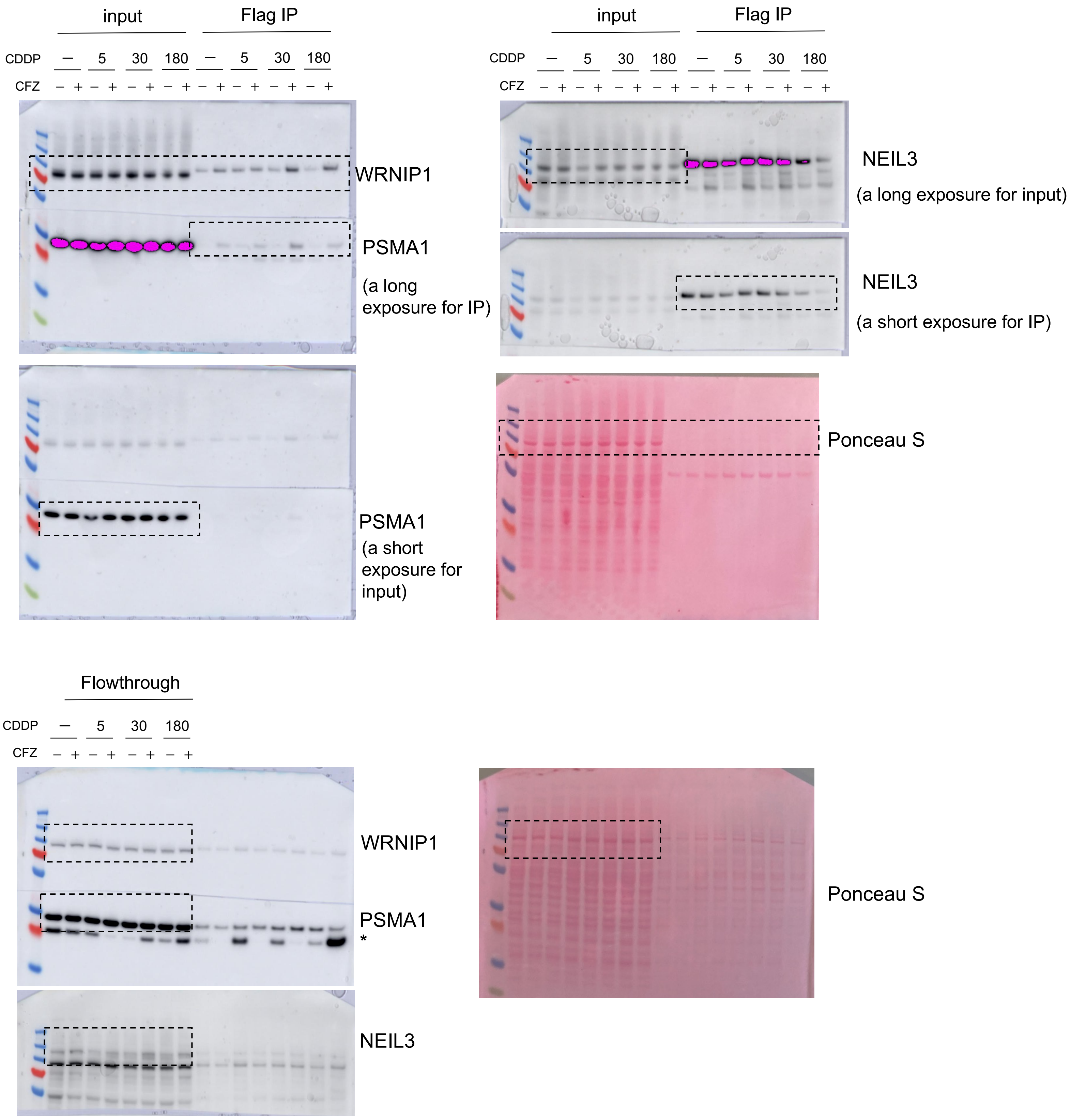

Figure S5. Full scan of the uncropped blots for Figure 3d. Membranes were cut at ~40kDa and incubated with different antibodies. Ponceau S staining was used as a loading control.

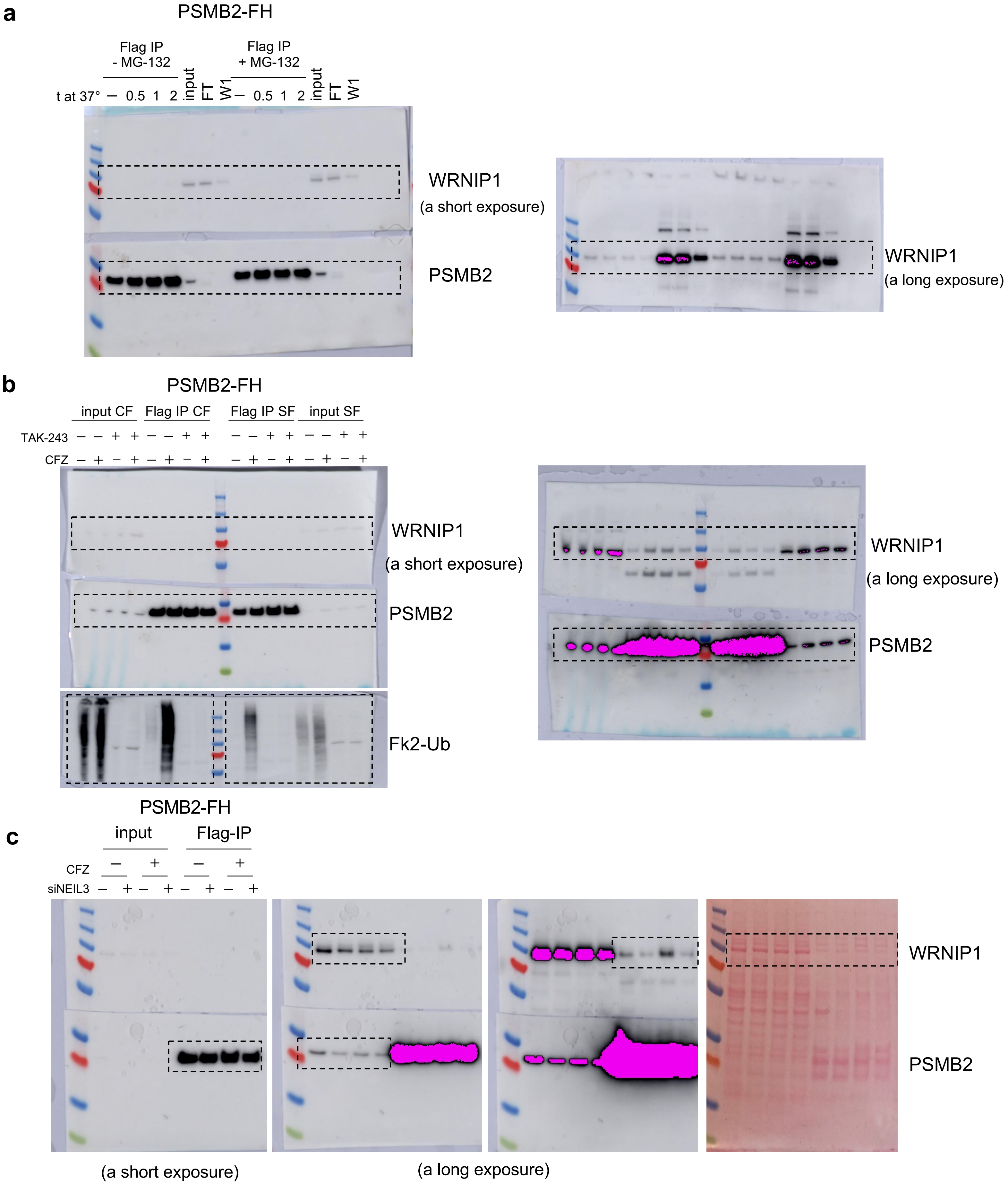

Figure S6. Full scan of the uncropped blots for Figure 4. (**a**) Original full scan blots for Fig. 4b. (**b**) Original full scan blots for Fig. 4c. (**c**) Original full scan blots for Fig. 4d. Membranes were cut at ~40kDa and incubated with different antibodies. Ponceau S staining was used as a loading control.

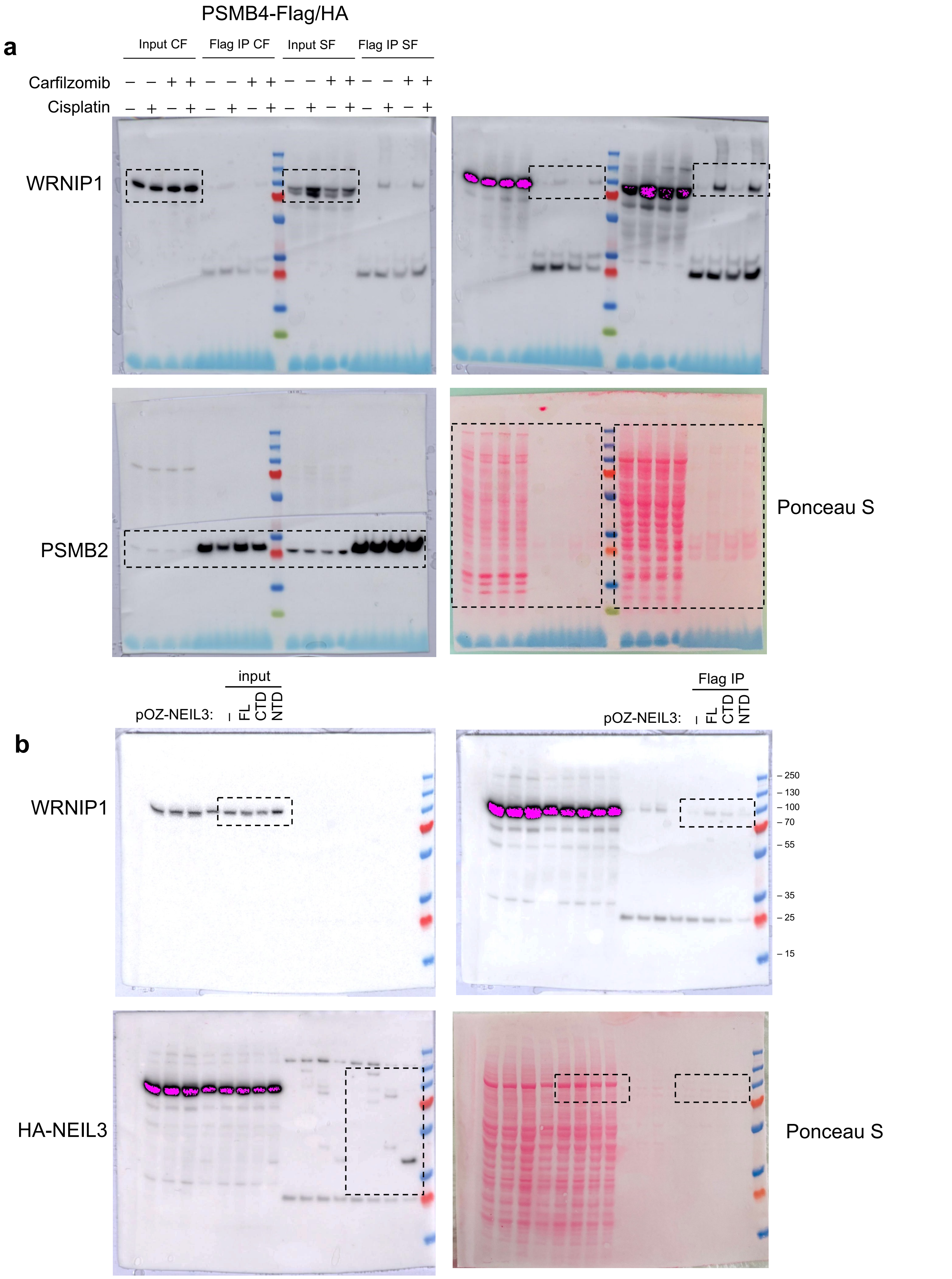

Figure S7. Full scan of the uncropped blots for Figures S1 and S3. (**a**) Original full scan blots from Fig. S2d. Membrane was cut at ~40kDa and incubated with different antibodies. (**b**) Original full scan blots for Fig. S2c.
